# Associations between serum and plasma brain-derived neurotrophic factor and influence of storage time and centrifugation strategy

**DOI:** 10.1038/s41598-019-45976-5

**Published:** 2019-07-04

**Authors:** Anne Kær Gejl, Christian Enevold, Anna Bugge, Marianne Skovsager Andersen, Claus Henrik Nielsen, Lars Bo Andersen

**Affiliations:** 1 University of Southern Denmark, Department of Sports Science and Clinical Biomechanics, Exercise Epidemiology, Odense, Denmark; 2 University College Copenhagen, Faculty of Health, School of Physiotherapy, Copenhagen, Denmark; 3 Copenhagen University Hospital Rigshospitalet, Institute for Inflammation Research, Center for Rheumatology and Spine Diseases, Copenhagen, Denmark; 4 Odense University Hospital, Department of Endocrinology, University of Southern Denmark, Odense, Denmark; 5 Western Norway University of Applied Sciences, Faculty of Education, Arts and Sport, Campus Sogndal, Bergen, Norway; 6 Norwegian School of Sport Sciences, Department of Sports Medicine, Oslo, Norway

**Keywords:** Blood proteins, Biological techniques, Biomarkers

## Abstract

The aims of the study were to clarify the impact of storage time and centrifugation strategy on brain-derived neurotrophic factor (BDNF) levels in human serum and plasma samples. In addition, we analyzed associations between BDNF levels, cardiorespiratory fitness and waist circumference. Seventeen healthy males (25.2 (4.1) years) were included in the study. Blood samples were drawn after an overnight fast and treated to different protocols, varying in time before centrifugation and centrifugation strategy. BDNF was analyzed in serum, normal plasma (NP) and platelet-poor plasma (PPP). Also, waist circumference and cardiorespiratory fitness were measured. A large increase was observed in serum BDNF levels during the first hour of clotting. BDNF in NP correlated with PPP, whereas no correlations were found between BDNF in serum and plasma. Though not statistical significant, correlations between fitness and BDNF in serum changed from positive at 30 min. to negative when clotting time was ≥60 min. In conclusion, BDNF levels in serum were affected by clotting time, whereas BDNF levels in plasma were influenced by centrifugation strategy. Importantly, BDNF in serum and plasma appears to reflect two different pools of BDNF. The biological relevance of the velocity of BDNF release during clotting and its dependence upon fitness must be investigated further.

## Introduction

Brain-derived neurotrophic factor (BDNF) is a growth factor protein and member of the neurotrophin family, which also includes, neurotrophin 3 and neurotrophin 4/5^1^. BDNF is widely distributed in the central nervous system (CNS)^2,3^, where it binds to the non-selective low-affinity necrosis factor receptor, p75^NTR^ and the high-affinity receptor tropomyosin-related kinase B (TrkB), and induces cell survival, -growth, and -differentiation as well as synaptic plasticity^1,4–6^. In resting adult rats high levels of BDNF have been identified in structures of the basal forebrain, the amygdala and the hippocampus^3^, and it is well accepted that BDNF plays an important role in the formation of memory and learning^7,8^.

In humans, BDNF levels in the brain (central BDNF) are difficult to measure, and therefore measurement of BDNF in the periphery is often used as a proxy. The relevance of this measure is supported by animal studies showing that BDNF is able to cross the blood-brain barrier in both directions^9^ and that peripheral and central BDNF levels are associated^10,11^. Although research in animals suggests that changes in circulating BDNF may be used as a proxy for changes occurring in the brain, application of different methodologies (e.g. biological medium, centrifugation strategy, temperature, and choice of bioassay) may introduce bias and complicate comparisons between studies. Peripheral BDNF is mainly stored in platelets (~99%), and only a small amount of free BDNF is present in plasma^12,13^. Since platelets cannot pass the blood-brain barrier, the BDNF level in the brain may not be reflected by the amount of BDNF associated with platelets, but rather by the amount of free BDNF in plasma^13^. Also, it is uncertain how precisely BDNF levels measured in venous blood samples reflects circulating BDNF levels *in vivo*, and how various methodological approaches affect the result of the analysis.

It is well-established that much higher concentrations of BDNF are observed in serum than in plasma^12,13^. In order to obtain serum, blood samples must coagulate prior to centrifugation. During the coagulation process activation of platelets causes a release of BDNF from platelets into serum^12^, suggesting that the length of the clotting time constitutes a critical methodological issue, when measuring the concentration of BDNF in serum^14–16^. Still, recommended clotting time varies greatly between different BDNF assays. Contrary to serum BDNF, plasma is obtained from blood samples drawn into tubes containing anti-coagulants, preventing coagulation and thus activation of platelets and BDNF release. One methodological study found no correlation between serum and plasma BDNF, suggesting that serum and plasma BDNF are independent measures of diverse biological relevance^15^. Due to the smaller amount of platelet-associated BDNF in plasma, BDNF measured in plasma may reflect the concentration of free BDNF. However, normal plasma contains platelets, which could potentially affect the amount of BDNF measured. To minimize any influence of platelet BDNF and thereby obtain a more genuine measure of freely circulating BDNF, some (e.g. Biosensis and R&D systems) but not all (e.g. Proteintech) manufacturers recommend a second centrifugation within 30 minutes of collection at ~10,000 g for 10 minutes. In contrast, other manufacturers do not recommend use of plasma for BDNF measurement (e.g. Aviscera bioscience), nor provide information about how to prepare the blood samples for plasma analyses (e.g. RayBiotech, Sigma-Aldrich). Despite obvious differences between serum and plasma BDNF, these measures are still used interchangeably in human literature.

Even though some methodological studies have been conducted in humans^14–19^, several methodological issues remain to be elucidated to enable construction of standardized and reliable protocols for assessment of peripheral BDNF and to enable a proper evaluation of the use of peripheral measures of BDNF in future studies. Therefore the primary objectives of the present study were 1) to determine how time between collection and centrifugation of blood samples as well as centrifugation strategy impact measured levels of peripheral BDNF (i.e. in serum and plasma), and 2) to determine any association between BDNF measured in serum, normal plasma (NP) and platelet-poor plasma (PPP). In addition, a large cohort study by Pedersen and colleagues reported that BDNF in serum was positively associated with cardiovascular risk factors^20^. Similar findings have been reported elsewhere^21–23^. In contrast, BDNF measured in plasma have been found to be positively associated with cardiorespiratory fitness^24,25^ and negatively with cardiovascular risk factors^25^. Such findings suggest that the application of various methodological approaches may influence the direction of these associations. Therefore, secondary objectives were to explore if BDNF levels assessed under different circumstances were associated with cardiorespiratory fitness and/or waist circumference, and the directions of these associations.

## Method

### Participants

Seventeen apparently healthy male students (mean age = 25.2 (4.1) years) from University of Southern Denmark, Odense, Denmark were included in the study. Exclusion criteria were (1) use of any type of medication excluding food/dietary supplements and (2) physical disabilities hindering participation in a maximal cycling test. No additional information about medical background was collected.

### Ethical approval

All participants provided written informed consent. No economic compensation was provided. The study was approved by The Regional Scientific Ethical Committee for Southern Denmark (S-20140105) and conform to the standards set by the Declaration of Helsinki, except for registration in a database.

### Procedure

After an overnight fast (minimum 8 hours), trained staff obtained blood from an antecubital vein (gauge of needle: 21 G x ¾″ × 7″, BD Vacutainer, REF 367282) into 15 vacutainers (five serum and 10 plasma samples). All samples were drawn at 8 a.m. and always in the same order. After blood drawing, anthropometric measures were assessed and a standardized breakfast was consumed. Approximately 1.5 hours after breakfast, a maximal cycling test was completed.

### Blood samples

Serum samples were left to clot at room temperature for 30, 60, 180, 240 or 300 min. Subsequently, all serum samples were centrifugated at room temperature for 15 min. at 1,000 g. Plasma samples were drawn into tubes containing ethylenediaminetetraacetic acid (EDTA) and immediately put on ice for 30, 60, 180, 240 or 300 min. All plasma samples were centrifugated at 4 °C for 15 min. at 1,500 g to obtain normal plasma (NP). A second centrifugation at 10,000 g for 10 min. (also at 4 °C) was completed for five plasma samples to obtain platelet-poor plasma (PPP). After centrifugation, samples were aliquoted into Eppendorf tubes and stored at −80 °C until analysis (~eight months). Samples were analyzed for mature BDNF (mBDNF) using the Mature BDNF Rapid ELISA kit (Cat.# BEK-2211) from Biosensis (Biosensis, Thebarton, Australia) according to the manufacturers instructions. The assay ranges from 7.8 to 500 pg/ml with intra-assay and inter-assay variations of ≤7.3% and ≤7.6%, respectively, and pro-BDNF cross-reactivity of approximately 5.3%; no cross-reactivity with other tested neurotrophins. BDNF levels below the quantitation limit of 156.25 pg/ml were given a value of 78.125 pg/ml. Potential influence of the variation between plates/kits was eliminated by ensuring that all samples from each participant were measured on the same plate/kit. Further, the room temperature during analyses ranged from 23.7–26.2 °C.

### Assay quality control

Initial analysis of five randomly selected samples suggested that a dilution factor of 1:400 was optimal for serum samples and a factor of 1:20 was optimal for plasma samples. At these dilutions, we saw mBDNF recovery rates of 85–110% for eight randomly selected samples spiked to a final 62.5 pg/ml. As mBDNF is derived from pro-BDNF, some cross-reactivity with pro-BDNF was expected, as stated by the manufacturer. In our hands, the assay showed approximately 25% cross-reactivity to pro-BDNF (i.e., a 10 pg/ml pro-BDNF spike translated to approximately 2.5 pg/ml mBDNF). However, since pro-BDNF levels are generally much lower than mBDNF levels, cross-reactivity was deemed negligible. The cross-reactivity of the Biosensis assay has also been evaluated in a previous study by Polacchini and colleagues^26^. As an additional control of assay performance, aliquots of an in-house serum sample was included in duplicates on all nine plate-runs yielding an average intra-assay CV of 2.8% (range: 0.4–6.4%) and an inter-assay CV of 7.6% with a mean mBDNF content of 20.4 ng/ml (range: 18.4–23.0 ng/ml).

### Cardiorespiratory fitness

Cardiorespiratory fitness tests (VO_2max_ in ml/kg/min) were conducted on an electronically braked cycle ergometer (Monark Ergomedic 839, Vansbro, Sweden) with oxygen uptake (VO_2_) measured directly using a calibrated AMIS 2001 Cardiopulmonary Function Test System (Innovision, Odense, Denmark). An initial five minute warm-up with a load of 110 Watts was completed, followed by increases of 40 Watts every two min. until exhaustion. The highest mean VO_2_ value over 30 seconds was used as VO_2max_. A cadence between 70–80 RPM was encouraged. The protocol has previously been used in healthy young individuals^27^. Heart rate was recorded every two min. throughout the test and at the time of exhaustion (Polar RS800CX, Polar Electro, Kempele, Finland). Maximal performance was achieved if three out of the following four criteria were reached: (1) respiratory exchange ratio >1.10, (2) heart rate > 185 bpm, (3) lactate value >8 mmol/L and/or 4) subjective approval by test administrator. All participants reached the minimum of three criteria. Cardiorespiratory fitness was calculated as VO_2max_ in milliliters divided by body weight in kilograms.

### Anthropometrics

Body weight was measured to the nearest 0.1 kg and stature was measured to the nearest 0.5 cm using standard equipment. Participants were dressed in shorts and without shoes. Body mass index (BMI) was calculated by dividing body weight (kg) with stature (m) squared. Waist circumference was measured at a level midway between the lowest rib and the cristae iliaca three times using anthropometric tape (Seca 201, Hamburg, Germany). The average of the two closest measurements was used.

### Statistics

Difference between serum BDNF levels at various time point were tested using repeated measures analysis of variance (ANOVA). Due to non-normal distribution of plasma BDNF data, differences between various time points within each sample type (i.e. BDNF in PPP and NP), were tested using Friedman test. Pairwise comparisons (serum) or separate Wilcoxon signed-rank tests (PPP and NP) were conducted on a priori selected combinations to examine changes in BDNF levels over time (i.e., 30 vs. 60 min., 60 vs. 180 min., 180 vs. 240 min. and 240 vs. 300 min.). In addition, differences in BDNF levels between sample types (serum, PPP & NP) at each time point were tested using Friedman test. Wilcoxon signed-rank tests were used to examine differences between various protocols (i.e., serum vs. PPP, serum vs. NP and PPP vs. NP). Spearman’s correlation was used to examine (1) associations between BDNF in PPP at 30 min. and BDNF in serum at all time points, (2) associations between BDNF in NP at 30 min. and BDNF in serum at all time points and (3) associations between BDNF in PPP at 30 min. and BDNF in NP and BDNF in PPP at all time points. Correlations between plasma BDNF levels (i.e. in PPP and in NP) and cardiorespiratory fitness and waist circumference, respectively, were tested using Spearman’s correlation, while correlations between BDNF in serum and cardiorespiratory fitness and waist circumference, respectively, were tested using Pearson’s correlation. Finally, the change in serum BDNF (velocity of release) was generated by subtracting serum BDNF at 30 min. from serum BDNF at 60 min., in order to examine whether the release of BDNF between 30 min. to 60 min. was related to cardiorespiratory fitness and/or waist circumference. No correction for multiple comparisons were made for any of the analyses.

Friedman tests and Wilcoxon signed-rank tests were performed in SPSS 24 (IBM SPSS Statistics 24, Armonk, 10504–1722 New York, United States), and the remaining analyses were performed using Stata 15.0 (StataCorp, College Station, Texas, USA). The level of statistical significance was set to *P* < 0.05. With n = 17, r > 0.456 becomes significant.

## Results

### Effects of time, centrifugation and sample type

Participant characteristics are presented in Table 1.

**Table 1:** Participant characteristics. Mean (SD) unless otherwise stated.

Variable	N = 17
Age (years)	25.2 (4.1)
Weight (kg)	78.7 (3.4)¤
Height (cm)	180.9 (6.6)
BMI (kg/m^2^)	23.7 (2.1)¤
VO_2max_ (L O_2_/min)	4.36 (0.5)
Fitness (mL O_2_/min/kg)	54.9 (7.3)
HR_max_ (bpm)	192.1 (9.0)
RPE (1–20)	19.4 (0.7)
Lactate (mmol/L)	13.2 (2.3)

^¤^Median (iqr). BMI = body mass index, VO_2max_ = maximal oxygen uptake, HR_max_ = maximal heart rate, RPE = rate of perceived exhaustion, bpm = beats per minute.

**Figure 1:**
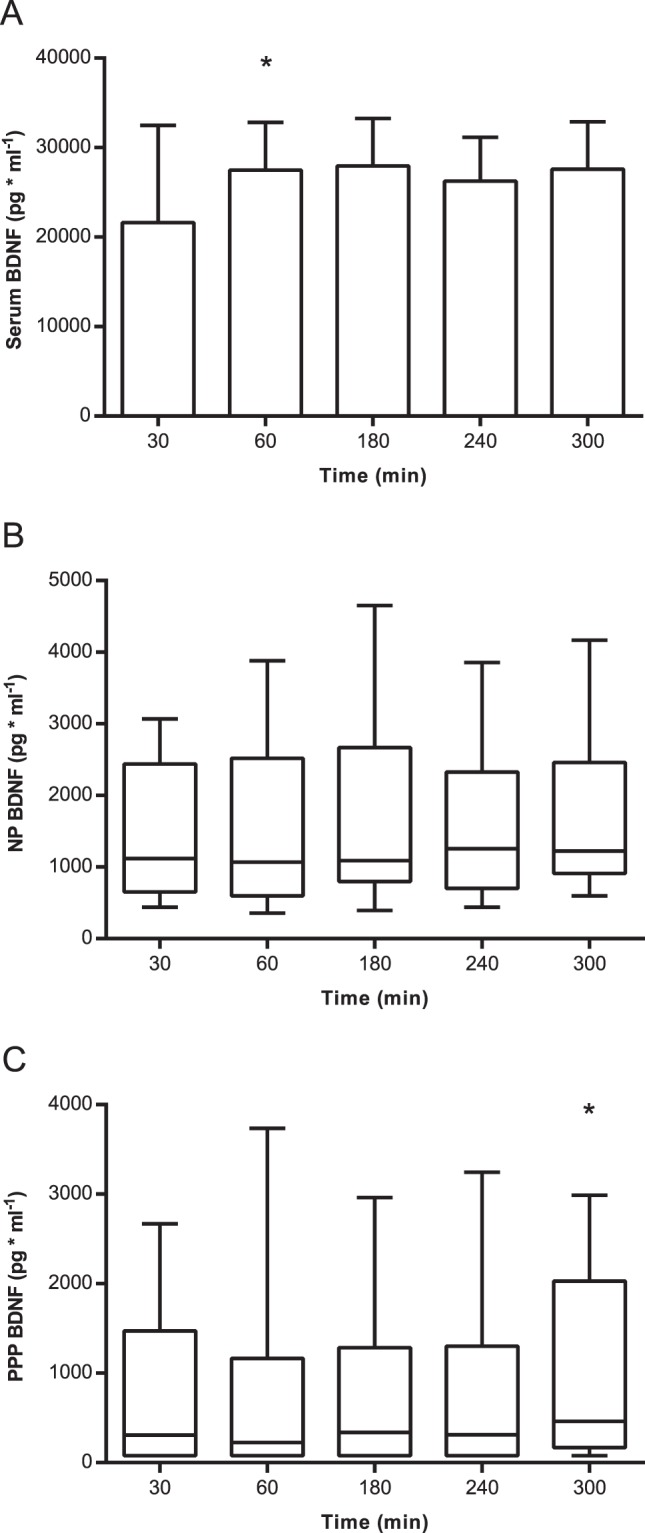
Mean (SD) for BDNF levels in serum (**A**), and median (IQR) for BDNF levels in normal plasma (**B**) and platelet-poor plasma. (**C**) An increase in BDNF levels measured in serum was observed between 30 and 60 min. (**A**) No systematic changes were observed in plasma BDNF over time (**B** & **C**). BDNF = brain-derived neurotrophic factor, PPP = platelet-poor plasma and NP = normal plasma. *Different from the preceding time point (*P* < 0.05).

**Figure 2:**
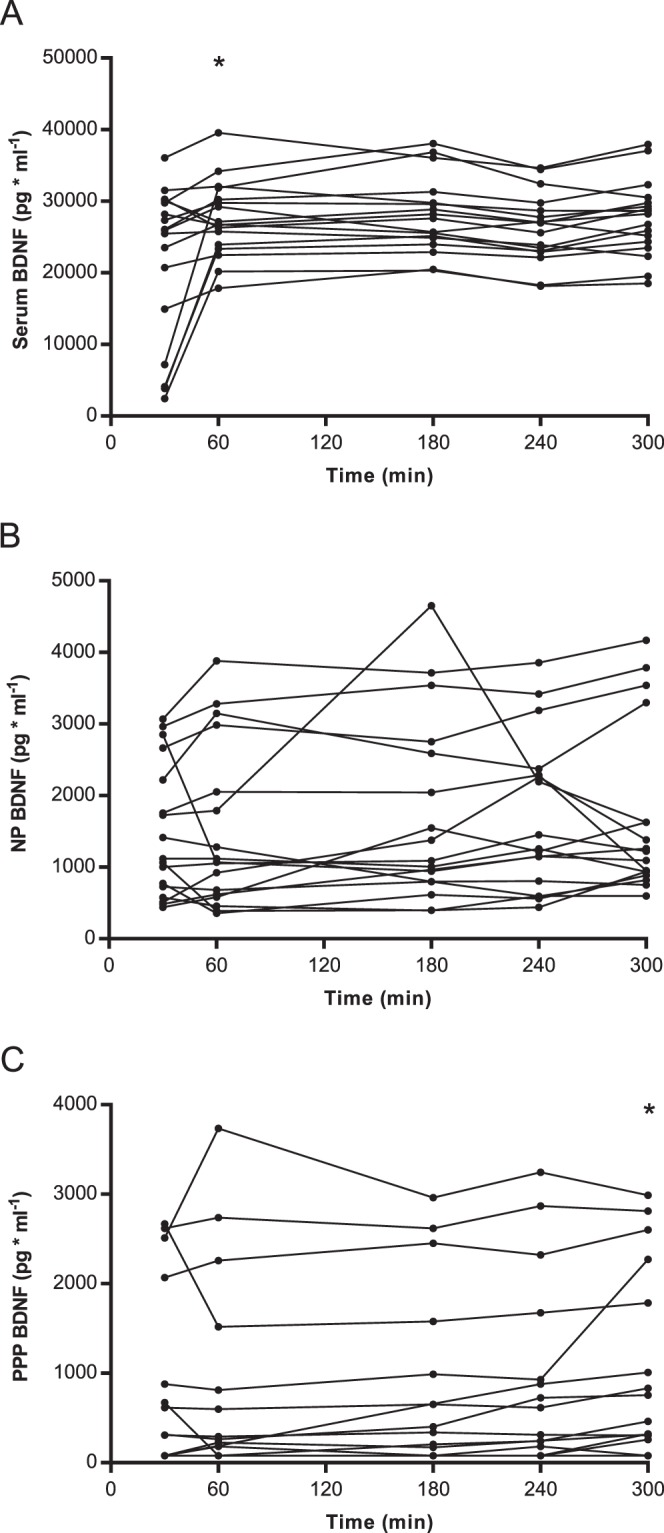
Individual levels of BDNF in serum (**A**), normal plasma (**B**) and platelet-poor plasma. (**C**) Great individual differences were observed in the initial release of BDNF into serum during coagulation. (**A**) Systematically higher BDNF levels were observed for NP BDNF compared to PPP BDNF (**B**,**C**). BDNF = brain-derived neurotrophic factor, PPP = platelet-poor plasma and NP = normal plasma. *Different from the preceding time point (*P* < 0.05).

Median and range for BDNF measured in NP and PPP and mean (SD) for BDNF measured in serum at the different time points are shown in Fig. 1, and individual levels are presented in Fig. 2. BDNF levels below the quantitation limit of 156.25 pg/ml were found in 34% of the PPP samples and were given a value of 78.125 pg/ml. At all time points, significant differences were observed between BDNF levels measured in serum, PPP and NP, with BDNF in serum > NP > PPP (all *P*s ≤ 0.004) (see Fig. 1).

We next examined how delay-time between collection and centrifugation of the blood samples influenced BDNF levels measured in serum, NP and PPP. An effect of time was observed for BDNF levels measured in serum, as well as for levels in PPP and NP (*P* < 0.001, *P* = 0.001 and *P* = 0.038, respectively). Although the difference in serum BDNF levels measured at 30 and 60 min. of clotting, varied greatly between individuals (Fig. 2A), post hoc analysis revealed a significant increase in BDNF levels at 60 min compared to those that clotted for only 30 min (Fig. 1). No differences were observed in BDNF levels when comparing the remaining serum samples over time (i.e. 60 vs. 180 min, 180 vs. 240 min or 240 vs. 300 min) (all *P*s > 0.05). No systematic changes in PPP or NP BDNF were found over time, and only the differences in PPP BDNF levels measured at 240 min and 300 min was statistically significant (*P* = 0.048) (Fig. 1B,C).

Correlation coefficients between BDNF levels in serum, PPP and NP are shown for all time points in Table 2. BDNF measured in PPP samples left for 30 min. correlated significantly with BDNF levels measured in PPP and NP samples for any time points (*P* ≤ 0.048); the exception being levels measured in NP samples at 180 min. (r_s_ = 0.468, *P* = 0.058). No correlations were observed between BDNF levels measured in serum and BDNF levels measured in NP or PPP obtained from samples left for 30 min., respectively (*P* ≥ 0.560 and *P* ≥ 0.111, respectively). However, though not significant negative r_s_-values (r_s_ between −0.40 and −0.28) were observed for correlations between BDNF analyzed in PPP samples left for 30 min. and BDNF levels measured in serum.

**Table 2:** Correlation coefficients between serum BDNF, PPP BDNF and NP BDNF at different time points, cardiorespiratory fitness and waist circumference.

	Serum 30 min	Serum 60 min	Serum 180 min	Serum 240 min	Serum 300 min	PPP 30 min	PPP 60 min	PPP 180 min	PPP 240 min	PPP 300 min	NP 30 min	NP 60 min	NP 180 min	NP 240 min	NP 300 min	Fitness	Waist
Serum 30 min	1.00	—	—	—	—	—	—	—	—	—	—	—	—	—	—	—	—
Serum 60 min	0.61*	1.00	—	—	—	—	—	—	—	—	—	—	—	—	—	—	—
Serum 180 min	0.45	0.90*	1.00	—	—	—	—	—	—	—	—	—	—	—	—	—	—
Serum 240 min	0.55*	0.95*	0.97*	1.00	—	—	—	—	—	—	—	—	—	—	—	—	—
Serum 300 min	0.57*	0.93*	0.91*	0.94*	1.00	—	—	—	—	—	—	—	—	—	—	—	—
PPP 30 min	−0.29	−0.40	−0.28	−0.29	−0.33	1.00	—	—	—	—	—	—	—	—	—	—	—
PPP 60 min	−0.10	−0.12	−0.03	−0.03	−0.01	0.82*	1.00	—	—	—	—	—	—	—	—	—	—
PPP 180 min	−0.10	−0.17	−0.09	−0.07	−0.05	0.78*	0.93*	1.00	—	—	—	—	—	—	—	—	—
PPP 240 min	−0.05	−0.12	−0.05	−0.02	0.01	0.76*	0.95*	0.99*	1.00	—	—	—	—	—	—	—	—
PPP 300 min	−0.16	−0.24	−0.15	−0.14	−0.14	0.81*	0.84*	0.95*	0.93*	1.00	—	—	—	—	—	—	—
NP 30 min	0.07	−0.15	−0.05	−0.02	−0.16	0.71*	0.66*	0.52*	0.55*	0.45	1.00	—	—	—	—	—	—
NP 60 min	0.04	−0.01	0.06	0.14	0.07	0.72*	0.85*	0.76*	0.81*	0.66*	0.83*	1.00	—	—	—	—	—
NP 180 min	0.40	0.31	0.34	0.42	0.36	0.47	0.68*	0.67*	0.72*	0.60*	0.52*	0.80*	1.00	—	—	—	—
NP 240 min	0.15	0.11	0.16	0.25	0.15	0.63*	0.77*	0.80*	0.80*	0.73*	0.61^*^	0.88*	0.91*	1.00	—	—	—
NP 300 min	0.45	0.12	0.17	0.22	0.14	0.49*	0.55*	0.63*	0.63*	0.64*	0.44	0.63*	0.87*	0.82*	1.00	—	—
Fitness	0.26	−0.22	−0.24	−0.21	−0.26	0.03	0.11	0.10	0.10	−0.02	0.29	0.08	−0.17	−0.08	−0.07	1.00	—
Waist	0.16	0.50*	0.52*	0.49*	0.58*	−0.15	0.10	0.07	0.11	0.02	−0.06	0.13	0.37	0.18	0.23	−0.66*	1.00

PPP = platelet-poor plasma, PRP = platelet-rich plasma, fitness = cardiorespiratory fitness (mLO_2_/min/kg), waist = waist circumference (cm).

### Correlation between BDNF, cardiorespiratory fitness and waist circumference

No statistically significant correlations were observed between cardiorespiratory fitness and BDNF levels measured in serum, PPP or NP, regardless of the time passed before centrifugation (*P* ≥ 0.256) (Table 2). Interestingly, the correlations of BDNF levels measured in serum changed from positive at 30 min. (r = 0.26) to negative at 60 min, 180 min. 240 min. and 300 min. (r = −0.26 to −0.21). In addition, a tendency toward a negative correlation was observed between cardiorespiratory fitness and the difference in BDNF levels measured in serum obtained from samples left to clot for 30 and 60 min., respectively (r = −0.456, *P* = 0.066). As such, the positive r-value between cardiorespiratory fitness and serum BDNF at 30 min which changed to a negative r-values at 60–300 min. indicate a fast initial release of BDNF from platelets in fit individuals followed by a lower total level after 60 min. of clotting.

Finally, positive correlation coefficients were observed between waist circumference and serum BDNF at 60, 180, 240 and 300 min (0.014 ≤ *P* ≤ 0.044) but not at 30 min. (*P* = 0.549). By contrast, waist circumference did not correlate with BDNF levels measured in PPP or NP, regardless of how long the samples were kept before centrifugation (*P* ≥ 0.142). No correlation was found between waist circumference and the difference in BDNF levels measured in serum obtained from samples left to clot for 30 and 60 min., respectively (r = 0.110, *P* = 0.674).

## Discussion

In this study, BDNF levels measured in serum samples increased significantly with time during the first hour between collection and centrifugation and became relatively stable thereafter. In agreement, Tsuchimine and colleagues observed that BDNF measured in serum increased during the first hour of coagulation at 25 °C and became relatively stable with a clotting time between one and 48 hours^15^. Maffioletti and colleagues reported that the largest increase in measured serum BDNF levels occurred between 10 and 30 min. of clotting and also found a plateau after 60 min.^14^. In contrast, a study by Amadio and colleagues observed no plateau after 60 min of clotting at room temperature, but a constant increase for 120 min.^16^. In general the majority of existing findings, but not all^16^, suggest that BDNF is rapidly released from platelets during coagulation at room temperature, and that most BDNF is released into serum within the first hour.

Notably, the direction of the association between BDNF in serum and cardiorespiratory fitness changed from positive at 30 min. to negative at 60 min., suggesting that the BDNF release into serum is dependent upon cardiorespiratory fitness. Assuming that BDNF levels are close to zero at time 0 min. (reflected by BDNF levels in PPP at 30 min.), such findings suggest a faster release in fit individuals. Circulating BDNF is upregulated in response to exercise, potentially due to its role in exercise-induced skeletal muscle regeneration^28^. A study by Cho and colleagues suggests that individuals with high cardiorespiratory fitness release more BDNF in response to acute aerobic exercise than individuals with lower cardiorespiratory fitness^29^. It can be speculated that not only the total amount of BDNF in serum and/or platelets is of biological relevance, but that rate of BDNF release is also important, and that it may be influenced by aerobic exercise. However, it is still unknown whether release of BDNF into serum during the clotting process reflects the platelets’ ability to release BDNF *in vivo*, when needed for muscle regeneration and/or reparation of additional tissue. This should be investigated further. Although BDNF levels measured in serum are suggested to reflect the amount of platelet-associated BDNF^12^, the biological relevance of an alteration of BDNF measured in serum and/or platelet is not clear. Importantly, associations between cardiorespiratory fitness and serum BDNF may be highly dependent upon, and potentially confounded by, the clotting time before centrifugation. It should be noted, however, that these analyses were exploratory and thus, the results must be confirmed in future studies.

Not surprisingly, higher levels of BDNF were observed in NP than PPP. Due to the second centrifugation (10,000 g/10 min.), PPP contains significantly less platelets than NP does^12^, which influences the levels of BDNF measured^30^. As such, BDNF levels measured in NP will be influenced by platelet-derived BDNF to a higher extend than the levels measured in PPP. Contrary to our findings, a study by Pareja-Galeano and colleagues showed no difference between BDNF levels measured in NP and PPP and concluded that the procedure to obtain PPP (second centrifugation) was not necessary^17^. Despite the exact same centrifugation strategies, discrepancies between our findings and theirs may be due to differences in time between blood collection and centrifugation, temperature, and the assay used to analyze BDNF.

In the present study, no effects of time between blood collection and centrifugation were observed for BDNF measured in PPP or NP. In contrast, Tsuchimine and colleagues concluded that BDNF measured in PPP increased over time, even when samples were kept at 4 °C^15^. However, they further demonstrated that the level of BDNF in PPP was dependent upon the anticoagulant used and the temperature. Changes over time were less pronounced for EDTA-treated plasma samples kept at 4 °C, conditions similar to those used in our study. The presence of anti-coagulant in the tubes should inactivate the platelets. However, studies report that blood drawn into EDTA-coated tubes contains platelets showing signs of activation and some degree of granule release^31^ and, consequently, BDNF levels in PPP may also to some extent be influenced by BDNF released from platelets after the collection of blood.

An enormous variability in plasma levels has been observed, ranging from ~100 to ~7000 pg/ml^32^. These inconsistencies may primarily be explained by large differences in the applied methodologies. We found strong correlations between BDNF levels measured in PPP left for 30 min. and BDNF levels measured in PPP or in NP at all time points except at 180 min., demonstrating a low intra-individual variation. As such, our findings suggest that studies using different protocols for analyzing relative levels of plasma BDNF may be compared, but comparisons of absolute values are problematic, since the levels measured in NP are higher than those measured in PPP.

In accordance with the literature^12^, we found that serum contained far higher concentrations of BDNF than plasma and observed no significant correlations between serum and plasma BDNF values. This is in line with previous findings^15,32^, but not all^18^, and suggests that serum BDNF and plasma BDNF are two independent measures, which could have diverse biological relevance.

Contrary to previous findings (see^33^ for review), no association between cardiorespiratory fitness and any of the BDNF levels were observed in the present study. However, since this was not the main focus of the study, the study may have insufficient power and too little inter-individual variation in cardiorespiratory fitness to detect such an association. Positive correlation coefficients were observed between waist circumference and BNDF measured in serum at all time points, except at 30 min. of clotting. Such findings may, in particular, be explained by the increased platelet activation observed in individuals with central obesity^34^. Moreover, platelet hyperactivity has been related to cardiovascular risk factors^35^ and low cardiorespiratory fitness^36^, which may contribute to the positive correlation observed between BDNF levels measured in serum and cardiovascular risk factors^20^ and the negative correlation between BDNF measured in serum and cardiorespiratory fitness^22,23^.

Despite a strong design, this study also have some limitation. Only healthy young men were included in the present study limiting the generalization of the findings to sexes, different age groups or subject suffering from various diseases. Further, the variations in cardiorespiratory fitness and waist circumference were small and the lack of associations to BDNF levels may be caused by lack of statistical power. However, still the directions of such associations should be explored. Further, in the present study the mature form of BDNF was measured and consequently, the influence of time between collection and centrifugation of blood samples and centrifugation strategy on pro-BDNF levels is still unknown. Further, the present study provides no information about the ratio between pro- and mBDNF and how this ratio may be moderated by various factors such as the preparation of blood samples. The binding of mature BDNF to its receptor (trkB) is found to trigger different signaling cascades^37^, promoting cell survival, -growth, and -differentiation as well as synaptic plasticity^1,4–6^. In contrast, the binding of pro-BDNF to its receptor (p75^NTR^) is related to neuronal cell death and synaptic withdrawal^37^. Due to these differing roles of pro- and mBDNF^38^ it is essential to differentiate between these two isotypes.

In conclusion, BDNF levels measured in peripheral blood are highly dependent upon the biological medium in which BDNF is analyzed, with 25–120 times higher concentrations in serum compared to plasma. BDNF levels measured in serum are affected by time between collection and centrifugation, whereas BDNF levels in plasma are influenced by the centrifugation strategy. Strong correlations were observed between BDNF measured in normal plasma and platelet-poor plasma (low intra-individual variation), suggesting that studies using different protocols for analyzing relative levels of plasma BDNF may be compared. Contrary, BDNF in serum and BDNF in plasma were not correlated and appears to reflect two different pools of BDNF. Based on these results, it is important that future studies, using peripheral measures of BDNF, consider the treatment of blood carefully, since the application of different methodological approaches may introduce bias and great inter-study variability. In addition, explorative results suggested that cardiorespiratory fitness is related to the BDNF release into serum during clotting. However, future studies are needed to elucidate the biological relevance of the velocity of BDNF release during clotting and its dependence upon cardiorespiratory fitness.

## Data Availability

Data is available upon request.
